# Catalog of triply periodic minimal surfaces, equation-based lattice structures, and their homogenized property data

**DOI:** 10.1016/j.dib.2023.109311

**Published:** 2023-06-14

**Authors:** Joseph W. Fisher, Simon W. Miller, Joseph Bartolai, Timothy W. Simpson, Michael A. Yukish

**Affiliations:** a Department of Mechanical Engineering, The Pennsylvania State University, University Park, PA 16802, USA; b Applied Research Laboratory, The Pennsylvania State University, State College, PA 16803, USA; c Department of Industrial and Manufacturing Engineering, The Pennsylvania State University, University Park, PA 16802, USA

**Keywords:** Additive manufacturing, 3D printing, Lattice structures, Elastic properties, Engineering design, Computer aided design, Lightweighting, DfAM

## Abstract

The use of lattice structure in the Design for Additive Manufacturing (DfAM) engineering practice offers the ability to tailor the properties (and therefore the response) of an engineered component independent of the material and overall geometry. The selection of a lattice topology is critical in maximizing the value of the lattice structure and its unique properties for the intended application. To support this, we have compiled a catalog of lattice structures from the literature that includes all Triply Periodic Minimal Surfaces (TPMS) for which a low-order Fourier series fit is known (so that they can be modeled and manufactured). We also include equations that do not directly correspond to known TPMS but do produce a triply periodic structure without sharp corners that would give rise to stress concentrations. This catalog includes images, elastic mechanical property data, and CAD models useful for the visualization, selection, and implementation of these lattice structures for any engineered structure.

**Specifications Table**

**Table:** 

Subject	Mechanical Engineering Computer Graphics and Computer-Aided Design	
Specific subject area	Design and characterization of lattice structures for additive manufacturing	
Type of data	• Equations (implicit models)	
	• Images	
	• Graphs	
	• Property data in .csv, .mat, and .h5 formatting	
	• Computer-Aided Design (CAD) files in .stl, .3mf, and .ntop formats	
How the data were acquired	• List of surfaces and equations taken from literature sources	
	• Images of lattices generated in nTopology using custom design blocks (provided)	
	• Critical and pinch level set values found using custom nTopology blocks and MATLAB scripts	
	• Property data, effective moduli plots, and anisotropy surfaces generated using in-house and available linear FEA [1] codes.	
Data format	• Raw	
	• Analyzed	
Description of data collection	The list of surfaces and equations was collected through manual search of available literature. When multiple equations were present in the literature for a surface, a goodness-of-fit criterion was used in MeshLab to select a recommended equation.	
Data source location	Institution: The Pennsylvania State University	
	City/Town/Region: University Park, PA	
	Country: USA	
	Primary data sources:	
	Sources for Implicit Equations	Original Sources for Several TPMS
	
	Wohlgemuth et al. [2]	Schoen [6]
	Von Schnering and Nesper [3]	Neovius [7]
	Hsieh and Valdevit [4]	Lidin and Larsson [8]
	MSRI’s Table of Surfaces [5]	Karcher [9]
Data accessibility	Repository name: Github - Equation-Based-Lattice-Structures-Dataset	
	Direct URL to data: https://github.com/jwf23/Equation-Based-Lattice-Structure-Dataset	

## Value of the Data

• This catalog represents a single source of data, descriptions, and definitions of dozens of TPMS structures commonly used in the engineering discipline with associated geometry, definitions, elastic properties, and other metrics useful to engineering design and material science. No such compilation exists in the literature.
• This catalog is of benefit to engineers, scientists, researchers, designers, and educators using TPMS for demonstration and application.
• When either designing lattices for a specific application or for experimentation, scientists, engineers, and designers can utilize this data in the selection and implementation of the lattice structures. They will be able to compare and generate TPMS-based lattices without having to develop the necessary code and workflow required.

## 1 Objective

We generated this dataset with two objectives in mind. First, to provide a comprehensive list of all TPMS-based lattice structures that can be modeled using equations found in the literature. Second, to generate and present self-consistent images, data, and 3D models for each of these lattice structures, enabling comparison and straightforward implementation by engineers, scientists, and designers.

## 2 Data Description

The database contains the following types of data:

• **Implicit surface equations:** Implicit equations approximating the TPMS are taken from literature (see Primary Data Sources), compared as needed, and presented.
• **Images of TPMS:** Produced from Surface Evolver files available on Ken Brakke’s website [10]. These images provide a high resolution and high accuracy representation of TPMS without using the implicit approximations. The surface is colored on both sides to match the two skeletal lattices in the images of the lattices. Surface Evolver files were only available for 11 of the 27 surfaces included in the catalog.
• **Images of lattices:** Images of the TPnS, TPSf, and TPxS derived from each TPMS are shown in color-coded (color-blind friendly) images separately and together.
• **nTopology files:** The nTopology files are implementations of the implicit surface equations that can output the TPxS, TPSf, and TPnS for a given lattice with variable cell size and volume fraction. They can be imported and implemented in designs, accelerating the integration of lattices in an application.
• **STL and 3MF files:** Triangle mesh files containing single unit cell examples of each lattice in STL and 3MF (with color) formats, generated using nTopology.
• **Graphs of volume fraction vs. level set:** The relationship between the level set used to generate a lattice and the volume fraction of the lattice is graphed. The volume fraction was determined by voxelizing the unit cell of the lattice and dividing the number of voxels in the lattice by the total number of voxels in the unit cell. The volume fraction was calculated for 200 linearly spaced points between the critical level set values. The curves of the volume fraction vs. level set plots are plotted in colors matching the lattice types shown in Table 1 and *§3: Images of Lattice Structures and Related TPMS*; sections of the curve where either the structure or the void space pinches off (becomes discontinuous) are denoted by a change of color and line type.

**Table 1:** Images of the Gyroid TPMS and lattices presented in the table format used in the consolidated data pdf. Each of the 27 surfaces in the dataset is given a row in the full table. The images are also provided separately in the repository.
• **Homogenization data:** The homogenization data for each lattice is presented in two formats:
  • Separate data files provided in both MATLAB (.mat) and hdf5 (.h5) file formats. These data files contain: level set, volume fraction, mesh resolution (reported as the number of voxels along the edge of the cubic unit cell), and homogenized constitutive matrix (these matrices have not been normalized). The first three are reported as n×1 vectors where n is the number of points calculated. The homogenized constitutive matrix is reported as a 6×6×n 3D matrix. Each 2D 6×6 matrix is the homogenized constitutive matrix relating to the same index n in the first three vectors. If the surface is congruent, there will be two sets of these four variables. If it is non-congruent it will have three sets. A Poisson’s ratio of 0.35 was used for the homogenization.
  • Graphs of the normalized $C_{11}$, $C_{12}$, and $C_{66}$ values of the homogenized constitutive matrix for human interpretation. The linear elastic response of each lattice is graphed separately as functions of volume fraction and level set. The graphs vs. volume fraction are overlaid with a representative anisotropy property surface at a volume fraction that is 20% of the way from the minimum to the maximum volume fraction of the specific lattice. The graphs vs. level set are included in the database, but not the summary document.
• **Comparison data for equation selection:** For the FRD, OCTO, P+C(P), Split–P, and K surfaces, multiple equations were found in the literature. The equations for each surface were compared using either: the Hausdorff Distance computed between mesh representation of the TPMS and the surface from each equation, or a statistical analysis of the surface’s mean curvature.
• **Comparison models for equation selection:** For the FRD and OCTO surfaces that were compared using the Hausdorff Distance, we include .3mf mesh files of the TPMS and surfaces from different equation that were compared. We also include processed mesh files that contain the TPMS mesh with color indicating either signed or unsigned Hausdorff Distance based on file name.

**Fig. 1:**
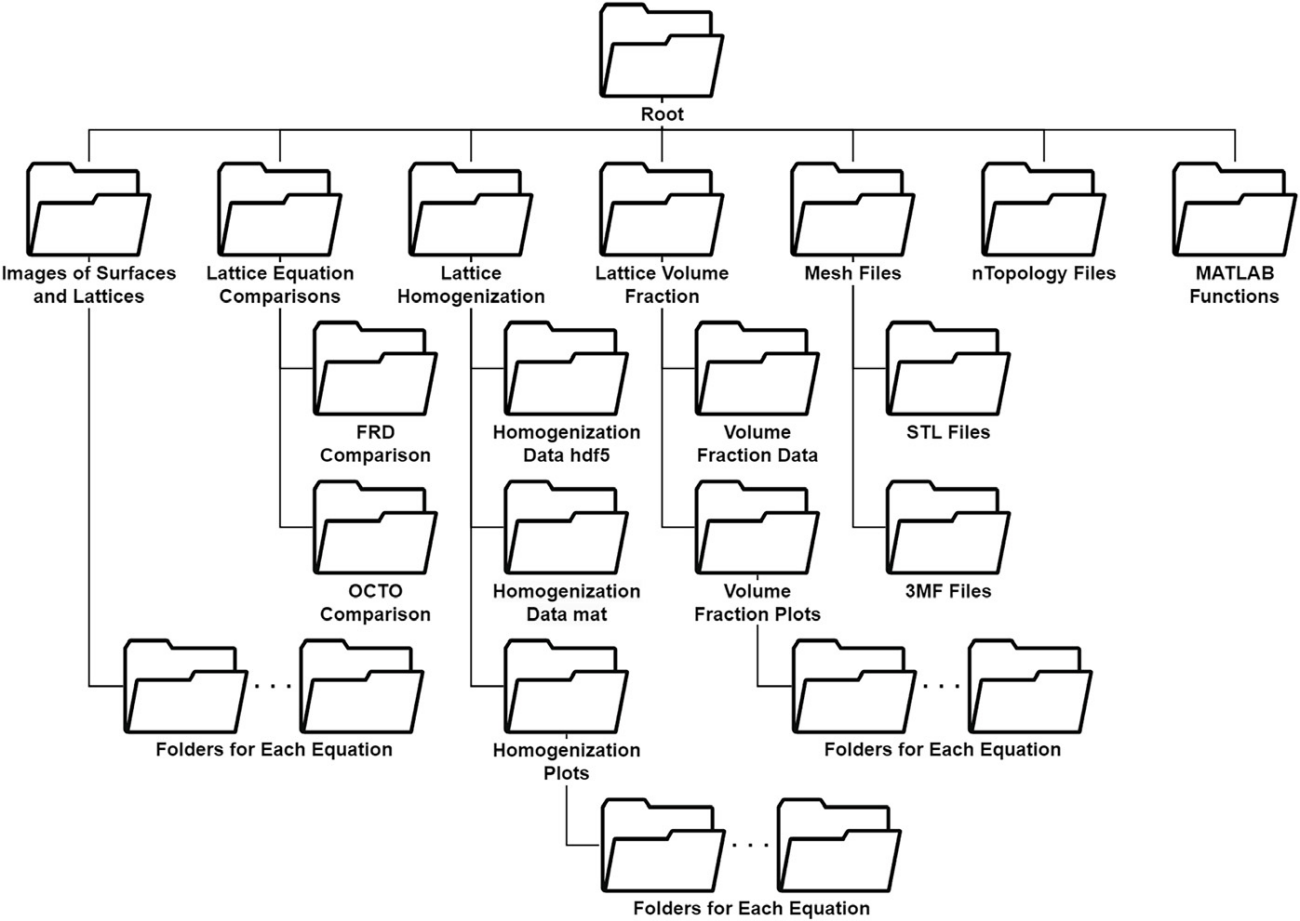
Folder structure of the GitHub repository.

Fig. 1 shows the file structure of the data repository. The aforementioned data is provided as separate files in the relevant folder as well as consolidated into a pdf (in the Root directory) for easier interpretation. Data is presented in the pdf in two ways. First, individual tables for each TPMS that bring together the equation, level set, and property data for its lattices (see Table 2). These tables are accompanied by any specific information about the TPMS or its lattices that is expected to be useful to the reader. Second, a table for visualization with each row dedicated to images of one surface and the lattices modeled off of it using the approximate implicit equations (see Table 1).

**Table 2:** Summary for the Gyroid taken from the consolidated data pdf. A similar table is provided for all 27 surfaces included in the dataset. All figures are included separately in the repository as well as the data used to generate them.

## 3 Experimental Design, Materials and Methods

The methods used to generate the variety of different types of data contained within this database are presented here.

The representations of the “true” TPMS are modeled in Surface Evolver by starting with a small fundamental region of the surface discretized into triangles and “evolving” it by subdividing the triangles of the surface and minimizing the energy due to surface tension subject to constraints on the boundaries. Through successive evaluations and refinement, it becomes a better and better approximation of the actual minimal surface. This was continued until the effect of the discretization was no longer visible at the resolution of the images. The unit cell is constructed by repeatedly reflecting the evolved fundamental region across its boundaries until a complete cell has been constructed.

The approximate implicit equation for each surface was implemented in nTopology and used to generate images and triangle meshes. Color was added to the mesh files in .3mf format using Microsoft’s 3D Builder [11].

The approximate implicit equations were also implemented in MATLAB and analyzed using a voxel-based linear FEA [1]. This code was used to produce volume fraction sweeps for each lattice which are presented in their respective summary tables (e.g. Table 2). The smooth curves on the effective elastic moduli plots were generated using a shape-preserving piece-wise cubic interpolation.^1^ In addition, this code estimated the upper and lower pinch and critical level sets. The reported values for the pinch and critical level sets were found by changing the level set value (limited to four significant digits) in nTopology until the limit was found.

The effective moduli plots shown in the summary tables are generated using the normalized $C_{11}$, $C_{12}$, and $C_{66}$ values from the homogenized constitutive matrix. In these plots, the curves are solid where the void regions are connected, and dash-dotted when the voids become discontinuous. Overlaid on each plot is a 3D visualization of anisotropy in the Young’s modulus of the lattice. These overplotted anisotropy surfaces give a snapshot at a single volume fraction and only for Young’s modulus; we elected to use a volume fraction that is 20% of the way from the minimum volume fraction where the lattice is connected to fully dense (VF=100%). This is marked on the three curves with black plus signs. The anisotropy surface is oriented to match the images of the surface and lattices in Table 1.

Where the literature provided multiple potential implicit equations and a Surface Evolver file exists, a comparison was performed between high-resolution mesh files exported from Surface Evolver for the TPMS and nTopology for the lattices. The metric used for goodness of fit was the unsigned Hausdorff Distance calculated using MeshLab [12]. If multiple equations were found but a Surface Evolver file was not available, the mean curvature of the implicit equations was calculated and the minimum, maximum, and average of the mean curvature were compared. The surface whose values were closer to zero was considered to be closer to *minimal*, which by definition has zero mean curvature at all points.

## Ethics Statements

Sources of curated data have been disclosed and no human subjects, animal experiments, or data collected from social media platforms were involved in this work.

## CRediT authorship contribution statement

**Joseph W. Fisher:** Data curation, Formal analysis, Methodology, Visualization, Writing – original draft. **Simon W. Miller:** Software, Writing – review & editing. **Joseph Bartolai:** Writing – review & editing. **Timothy W. Simpson:** Writing – review & editing. **Michael A. Yukish:** Writing – review & editing.

## Declaration of Competing Interest

The authors declare that they have no known competing financial interests or personal relationships that could have appeared to influence the work reported in this paper.

## Data Availability

Equation-Based-Lattice-Structure-Dataset (Original data) (GitHub).

## Footnotes

1 Using the pchip method implemented in MATLAB’s interp1 function.
